# Antenatal Atazanavir: A Retrospective Analysis of Pregnancies Exposed to Atazanavir

**DOI:** 10.1155/2014/961375

**Published:** 2014-09-25

**Authors:** Miriam Samuel, Daniel Bradshaw, Melissa Perry, Sum Yee Chan, Rageshri Dhairyawan, Laura Byrne, Katherine Smith, Judith Zhou, Charlotte Eve Short, Claire Naftalin, Ngozi Offodile, Sundhiya Mandalia, Sherie Roedling, Rimi Shah, Gary Brook, Mary Poulton, Mette Rodgers, Liat Sarner, Heather Noble, Philip Hay, Jane Anderson, Macky Natha, David Hawkins, Graham Taylor, Annemiek de Ruiter

**Affiliations:** ^1^Guy's and St Thomas' NHS Foundation Trust, Westminster Bridge Road, London SE1 7EH, UK; ^2^Chelsea and Westminster NHS Foundation Trust, 369 Fulham Road, London SW10 9NH, UK; ^3^St George's Healthcare Trust, Blackshaw Road, London SW17 0QT, UK; ^4^Homerton University Hospital NHS Foundation Trust, Homerton Row, London E9 6SR, UK; ^5^Newham University Hospital NHS Trust, Glen Road, London E13 8SL, UK; ^6^Barts and the London NHS Trust, West Smithfield, London EC1A 7BE, UK; ^7^Croydon University Hospital NHS Trust, London Road, Surrey CR7 7YE, UK; ^8^Imperial College Healthcare NHS Trust, Praed Street, London W2 1NY, UK; ^9^King's College Hospital NHS Foundation Trust, Denmark Hill, London SE5 9RS, UK; ^10^North West London Hospitals NHS Trust, Acton Lane, London NW10 7NS, UK; ^11^Mortimer Market Centre, Central and North West London NHS Foundation Trust, Capper Street, London WC1E 6JB, UK; ^12^Barnet and Chase Farm Hospitals NHS Trust, Wellhouse Lane, Barnet EN5 3DJ, UK

## Abstract

*Introduction.* There are few data regarding the tolerability, safety, or efficacy of antenatal atazanavir. We report our clinical experience of atazanavir use in pregnancy.* Methods.* A retrospective medical records review of atazanavir-exposed pregnancies in 12 London centres between 2004 and 2010.* Results.* There were 145 pregnancies in 135 women: 89 conceived whilst taking atazanavir-based combination antiretroviral therapy (cART), “preconception” atazanavir exposure; 27 started atazanavir-based cART as “first-line” during the pregnancy; and 29 “switched” to an atazanavir-based regimen from another cART regimen during pregnancy. Gastrointestinal intolerance requiring atazanavir cessation occurred in five pregnancies. Self-limiting, new-onset transaminitis was most common in first-line use, occurring in 11.0%. Atazanavir was commenced in five switch pregnancies in the presence of transaminitis, two of which discontinued atazanavir with persistent transaminitis. HIV-VL < 50 copies/mL was achieved in 89.3% preconception, 56.5% first-line, and 72.0% switch exposures. Singleton preterm delivery (<37 weeks) occurred in 11.7% preconception, 9.1% first-line, and 7.7% switch exposures. Four infants required phototherapy. There was one mother-to-child transmission in a poorly adherent woman.* Conclusions.* These data suggest that atazanavir is well tolerated and can be safely prescribed as a component of combination antiretroviral therapy in pregnancy.

## 1. Introduction

Atazanavir has been licensed for the treatment of HIV infection in nonpregnant adults in the UK since 2004. Off-license antenatal use is increasing; however, safety and efficacy data in this setting are lacking.

Atazanavir is a protease inhibitor (PI), a drug class which has been associated in some reports with increased risk of preterm delivery (PTD) and gestational diabetes [1–6]. Inhibition of uridine diphosphate-glucuronosyl transferase (UGT) 1A1 enzyme by atazanavir results in an unconjugated hyperbilirubinemia often presenting as jaundice; some have questioned whether this can result in increased neonatal hyperbilirubinemia [7, 8]. The optimum dosing regimen for atazanavir in pregnancy is also debated. The standard atazanavir-containing regimen consists of atazanavir 300 mg given with ritonavir 100 mg once daily (QD) in combination with two nucleos(t)ide reverse transcriptase inhibitors (NRTIs); ritonavir boosts plasma atazanavir concentrations through cytochrome P450 inhibition. Atazanavir can be administered without ritonavir at an increased dose; however this is not recommended in pregnancy. Some authors advise increasing the atazanavir dose to 400 mg QD as both coadministration of tenofovir [9, 10] and antenatal physiological changes are associated with reduced total plasma atazanavir concentrations [10].

Safety concerns prevent the inclusion of pregnant women in clinical trials; therefore, observational data are required to assess the safety and efficacy of novel agents antenatally. The aim of this study is to report maternal and neonatal outcomes following antenatal atazanavir exposure in the routine clinical setting.

## 2. Methods

This was a retrospective medical records review of pregnancies exposed to atazanavir and completing 12 weeks' gestation across 12 participating London HIV specialist centres between March 1, 2004, and December 1, 2010. Data were abstracted from medical records on demographics; medical history; symptoms; CD4+ T-lymphocyte counts (CD4 counts); HIV viral load (VL); treatment; adherence; maternal biochemistry; pregnancy and infant outcomes; neonatal bilirubin; and infant HIV infection status.

The indication for atazanavir prescription was categorised for each pregnancy as “preconception,” if they were already taking an atazanavir-containing regimen at the time of last menstrual period (LMP); “switch,” if atazanavir was commenced during this pregnancy in a woman already taking combination antiretroviral therapy (cART); “first-line,” if a first-line atazanavir-based regimen was initiated after the LMP. The “switch” group included women conceiving on, or initiating during this pregnancy, a non-atazanavir-based cART regimen. Women were classified as discontinuing atazanavir if a clinical decision was made to discontinue atazanavir prescription prior to the end of pregnancy.

In the UK, maternal HIV infection is diagnosed in accordance with national guidelines, by detection of HIV-specific antibodies using a commercial enzyme-linked immunoassay (EIA). The initial reactive assay is confirmed by a further, different, and type-specific EIA. The diagnosis of HIV infection is reconfirmed on a separate sample taken at a different time point [11]. Women testing positive for HIV 2 antibodies were excluded from the analysis. Hepatitis B virus (HBV) or hepatitis C virus (HCV) coinfection was diagnosed by the detection of antigen (HBV) or the respective nucleic acids at any point during the pregnancy. Hepatotoxicity was defined according to the AIDS Clinical Trial Group (ACTG) grading of grade 1–4 transaminitis [12]. Plasma HIV-1 RNA <50 copies/mL was classified as “undetectable” and ≥50 copies/mL as “detectable.”

Mode of delivery was classified as vaginal delivery (VD), planned prelabour prerupture of membranes Caesarean section (PLCS), or emergency Caesarean section (ECS), the latter including all unplanned Caesarean sections. Analysis of birth weight and gestational age at delivery included data only from singleton deliveries. Deliveries at <37 weeks' completed gestation were classified as spontaneous preterm if labour occurred spontaneously or iatrogenic preterm in the event of induced delivery or PLCS. Neonates weighing <2500 g were classified as having low birth weight (LBW). Mother-to-child transmission was identified through testing infants for HIV proviral DNA at birth and at >3 months of life, as per the British HIV Association Guidelines [13]. Peak neonatal bilirubin concentrations measured within the first 14 days of birth were recorded. Neonatal hyperbilirubinemia was classified as per the ACTG grading [14]. Use of phototherapy was recorded. Congenital (birth) defects as defined by the European Surveillance of Congenital Abnormalities (EUROCAT) [15] were reported if identified antenatally through screening programmes or during the neonatal examination.

Anonymised data were entered into a specifically designed Access database. Once cleaned the data were exported to STATA v12 for statistical analyses. Quantitative data with normal distribution are presented as means with 95% confidence intervals (CI) while non normal data are presented as medians with interquartile range (IQR). For the purpose of analysis each pregnancy was treated as a case. A logistic regression model was used to assess the strength of associations between different exposures and HIV-VL <50 copies/mL at delivery both in a univariate analysis and after adjustment for HIV-VL at the first antenatal visit and whether atazanavir had been prescribed preconception, first-line, or switch. The following methods were used to account for the correlation caused by multiple pregnancies occurring in the same woman: robust standard errors and generalised estimating equations (GEE) with an exchangeable correlation matrix to model clustered data. All *P* values presented are two-sided and generated by Wald-type testing.

The use of anonymised clinical data collected as part of routine care for this evaluation was conducted in accordance with the UK National Research Ethics Service (NRES) guidance.

## 3. Results

There were 145 pregnancies in 135 women. All were HIV-1 positive. None were HIV-2 positive. Median age at the first antenatal appointment was 32 years (IQR 28–37 years). Recorded ethnicities were Black-African, 110 (75.9%); Black-Caribbean, 10 (6.9%); and White, 8 (5.5%). HIV transmission risks were heterosexual intercourse, 136 (93.8%); injecting drug use, 5 (3.4%); mother-to-child-transmission, 3 (2.1%); and blood transfusion, 1 (0.7%). Seven pregnancies (4.8%) occurred in women who were HBV-coinfected and two (1.4%) in women who were HCV-coinfected. Median maternal CD4 count at first antenatal appointment was 407 (range: 15–1161) cells/mm^3^. Categories of atazanavir exposure were as follows: preconception, 89 (61.4%); switch, 29 (20%); first-line, 27 (18.6%). There was no significant difference in the demographic composition of the different atazanavir exposure groups. Data on prevalence of nausea and vomiting, new-onset transaminitis, VL at delivery, PTD, birth weight, neonatal jaundice, and events of mother-to-child-transmission are summarised by exposure group in Table 1. Key details are presented for each exposure group below.

### 3.1. Preconception Atazanavir

Of the 89 women who received preconception atazanavir, five discontinued atazanavir prior to delivery: two due to teratogenicity concerns, two due to nausea, and one due to virological failure. Three women were coinfected with HBV and two were coinfected with HCV. There was one case of ACTG grade 2 transaminitis in a woman coinfected with HCV; atazanavir was continued in this case and the transaminitis resolved spontaneously prior to delivery.

There were two intrauterine deaths: one at 14 weeks' gestation associated with trisomy 21 (maternal age 39) and one stillbirth at 27 weeks' gestation, the cause of which is unclear and no autopsy results are available. There were 82 live births amongst women who conceived on atazanavir (80 singleton and 2 twin deliveries), amongst which there were two reported congenital abnormalities: one case of trisomy 21 with an associated atrial septal defect (maternal age 44) and one severe congenital cardiac abnormality incompatible with life (maternal age 38). There were no ACTG grade 2–4 events of neonatal hyperbilirubinemia. Phototherapy was used in one twin with haemolytic disease of the newborn (neonatal bilirubin 194 *μ*mol/litre). One infant, delivered by ECS, was infected* in utero,* with HIV proviral DNA detected at delivery and at age three months. Maternal adherence to antiretroviral therapy during pregnancy had been poor and HIV-VL at delivery was 1103 copies/mL. No other HIV transmissions were reported. A negative HIV proviral DNA result was available at delivery and at 3 months for 73 and 67 infants, respectively.

### 3.2. First-Line Atazanavir

Of the 27 women receiving first-line atazanavir, three discontinued atazanavir prior to delivery: two for nausea and vomiting and one for abdominal pain and gallstones. Nausea and vomiting were reported in 15 (55.6%; 95% CI 35.5–75.6%).

Two cases were coinfected with HBV; none were coinfected with HCV. All 27 women commencing first-line atazanavir did so with normal baseline transaminase levels. Spontaneously resolving transaminitis developed in 3 (11%) cases (ACTG grade 2 in 2 cases who were not HBV/HCV-coinfected and ACTG grade 4 in 1 case with HBV coinfection).

There were no intrauterine deaths or reports of congenital abnormalities. Atazanavir was continued to delivery in 23 singleton and one twin pregnancies. There were no ACTG grade 2–4 events of neonatal hyperbilirubinemia. Two singleton infants with comorbidities required phototherapy. These infants had been diagnosed with polycythaemia neonatorum (neonatal bilirubin 258 *μ*mol/litre) and infant haemolytic anaemia (neonatal bilirubin 109 *μ*mol/litre), respectively. The median duration of antiretroviral treatment prior to delivery was 16.8 weeks (range: 2.7–30.4 weeks). Overall, an undetectable VL at delivery was achieved in 13/23 women who were prescribed first-line atazanavir (56.5%, 95% CI 34.8–76.0%). When categorised by the duration of ART exposure prior to delivery, an undetectable viral load was achieved in 4/10 women who received <16 weeks cART (40.0%, 95% CI 10.2–63.5%) compared to 9/13 who received ≥16 weeks cART (69.2%, 95% CI 36.5–89.8%). HIV proviral DNA results were available for all 24 infants at delivery and for 19 singletons at three months, with no reported cases of mother-to-child-transmission.

### 3.3. Switch Exposure to Atazanavir

Of the 29 women who switched to atazanavir, 13 had conceived on an alternative regimen and 16 had commenced an alternative regimen during the course of this pregnancy. Three women discontinued atazanavir prior to delivery: one due to intolerance and two due to transaminitis.

The commonest reason for switching to atazanavir was symptomatic intolerance of the discontinued regimen: 21/29 switched because of gastrointestinal symptoms attributed to antiretroviral therapy. Of the 20 who switched from another PI, symptoms improved in 19 (95.0%, 95% CI 69.6–98.8%). Ten of the 29 women (34.5%; 95% CI 16.1–52.9%) had new or worsening of gastrointestinal side effects after switching to atazanavir which was also discontinued in one pregnancy.

Two cases were coinfected with HBV; none were coinfected with HCV. Transaminase levels were normal at the time of atazanavir switch in 24/29 cases; none of these developed new-onset transaminitis (Table 1). In five cases atazanavir was commenced in women with elevated baseline transaminases. In two of these five cases, atazanavir was discontinued due to persistent transaminitis; in both cases women had switched to an atazanavir-based regimen from a lopinavir-based regimen with grade 2 transaminitis, following which atazanavir was switched to raltegravir with subsequent resolution of transaminitis. Neither case was coinfected with HBV or HCV. Atazanavir was continued with resolution of transaminitis in three of these five cases, of which two, who were both HBV-coinfected, had switched from lopinavir-containing therapy because of nausea and grade 3 transaminitis and one had switched from nevirapine-containing therapy because of hypersensitivity and grade 1 transaminitis.

No intrauterine deaths or congenital abnormalities were reported in this group. Excluding the three cases in which atazanavir was discontinued, there were 26 live singleton deliveries. There were no ACTG grade 2–4 events of neonatal hyperbilirubinemia. One infant with no recorded comorbidities required phototherapy (neonatal bilirubin 194 *μ*mol/litre). HIV proviral DNA results were available for 24 infants at delivery and 22 infants at 3 months, with no reported cases of mother-to-child transmission.

### 3.4. Atazanavir Dosing Regimens and Virological Suppression at Delivery

Data on HIV-VL at delivery amongst women prescribed different atazanavir dosing regimens are displayed in Table 2. Women who remained on atazanavir 300 mg/ritonavir 100 mg throughout pregnancy were most likely to achieve an undetectable HIV-VL at delivery; however there was no good evidence of an association between undetectable VL at delivery and the atazanavir dosing regimen in the univariate (*P* = 0.1) or multivariate analysis (*P* = 0.1) after adjusting for atazanavir treatment group and HIV-VL at the first antenatal visit.

## 4. Discussion

We present data on outcomes seen amongst women who have been prescribed atazanavir in a routine clinical environment. Overall atazanavir was well tolerated, with hepatotoxicity necessitating treatment switch occurring only when atazanavir was commenced on a background of existing transaminitis. Neonatal hyperbilirubinemia requiring phototherapy was uncommon and the only mother-to-child transmission event was attributed to poor adherence.

Large HIV cohort studies such as The Antiretroviral Pregnancy Register [16] and the National Study of HIV in Pregnancy and Childhood [17] continue to report on many antenatal outcomes including antiretroviral teratogenicity, virological efficacy, mother-to-child transmission, and trends in antenatal antiretroviral use. However, these studies do not capture data on some clinically relevant queries such as drug tolerability, toxicity, or optimum drug dosing. Our analysis focuses on these questions.

Intolerance was the most common indication for switching to atazanavir following which symptoms resolved in the majority. In the absence of a control group we cannot conclude that symptom resolution was attributable to switching. Our findings do suggest, however, that atazanavir may be a useful option to consider amongst women intolerant of other regimens. This is in line with the findings of a recently published observational analysis which suggests that atazanavir-based therapy is comparable to lopinavir-based therapy in terms of safety and efficacy [18].

These data do not support routine atazanavir dose escalation in pregnancy, in keeping with another recent report [19]. Despite most women remaining on atazanavir 300 mg/ritonavir 100 mg during the third trimester, the overall rate of complete HIV viral load suppression at delivery and the rate of mother-to-child transmission amongst those who conceived on atazanavir were comparable to those reported in other cohorts [20–24]. A low percentage of women who commenced first-line atazanavir achieved an undetectable HIV viral load at delivery (56.5%); however 10/23 of this group had received less than 16 weeks of cART prior to delivery, a shorter treatment duration than that recommended by the British HIV Association [13].

The neonatal outcomes are reassuring in terms of PTD and neonatal jaundice. The risk of PTD compares favourably to rates reported with other cART- and specifically PI-exposed cohorts [2, 25–27]. The need for phototherapy (4/120) was much lower than that reported in another observational cohort where 5/23 neonates required phototherapy [8]. However, our findings are in keeping with other published data which support the theory that* in utero* exposure to atazanavir does not exacerbate physiological jaundice [28]. This may be attributable to poor transplacental atazanavir transfer [29].

The main limitations to this study are selection bias and information bias: due to the limited experience of atazanavir in pregnancy, the women in our study partly represent a cohort that was intolerant of other more established regimens (the switch cohort), whilst the indications for commencing first-line atazanavir were not recorded. As with all medical records reviews, data collection was retrospective and dependant on accuracy and availability of clinical record keeping. This has resulted in large amount of missing data which could introduce bias into the analysis, especially affecting infant outcomes. The follow-up of* in utero* exposed infants is limited to neonatal examination and postnatal HIV testing; we therefore cannot comment on whether* in utero* atazanavir exposure is associated with longer-term risks to the infant. In addition, despite the size of the overall cohort, the small sample size of subgroups limits the precision of estimates in subgroup analysis.

## 5. Conclusion

This is, to our knowledge, the largest case series to date of atazanavir use in pregnancy. In summary we found that atazanavir as part of antenatal cART appears safe and well tolerated. Data on HIV viral load at delivery and transmission have not raised any concerns regarding efficacy for women conceiving on or switching to atazanavir-based cART. The overall risk of mother-to-child transmission is similar to that reported in other cohorts. Further data are required to assess whether initiation of atazanavir-based cART as a first line regimen during pregnancy has a similar efficacy in achieving virological suppression by the time of delivery in comparison with other antiretroviral regimens.

## Figures and Tables

**Table 1:** Birth outcome data for pregnancies exposed to an atazanavir (ATV) containing regimen. Data are presented based on the indication for atazanavir use.

		Indication for atazanavir use∗					
		Preconception		First-line		Switch	
Total pregnancies∗∗	*n* ^‡^		89		27		29
ATV discontinued	*n*, % (95% CI)^‡‡^		5, 5.6% (1.8–12.6%)		3, 11.1% (2.4–29.1%)		3, 10.3% (2.2–27.4%)
Gastrointestinal side effects∗∗∗	*n*, % (95% CI)		23, 25.8% (16.5–35.1%)		15, 55.6% (35.5–75.6%)		10, 34.5% (16.1–52.9%)
New-onset transaminitis^†^	*n*, % (95% CI)		1, 1.1% (0.02%–6.1%)		3, 11.0% (2.4–29.1%)		0^††^, 0% (0–14.2%)
Singleton deliveries^†††^	*n*, % (95% CI)		80, 97.6% (91.5–99.7%)		23, 95.8% (78.9–99.9%)		26, 100% (86.8–100%)
Twin deliveries^†††^	*n*, % (95% CI)		2, 2.4% (0.3–8.5%)		1, 4.2% (0.1–21.1%)		0 (0–13.2%)
Gestation <37 weeks^§^	*n*, % (95% CI)		9, 11.7% (5.5–21.0%)		2, 9.1% (1.1–29.2%)		2, 7.7% (1.0–25.1%)
Birth weight (grams)^§^	Median (IQR)	N^‡‡‡^ = 65	3160 (2870–3360)	*N* = 21	3030 (2720–3255)	*N* = 23	3030 (2670–3480)
Low birth weight (<2500 grams)^§^	*n*, % (95% CI)		6, 9.2% (3.5–19.0%)		3, 14.3% (3.0–36.3%)		3, 13.0% (2.7–33.6%)
Neonatal bilirubin (*μ*mol/litre)	Median (IQR)^§§^	*N* = 54	70.5 (45.0–107.0)	*N* = 16	76 (54.3–112.3)	*N* = 20	70.5 (49.8–88.8)
HIV viral load <50 (copies/mL)	*n*, % (95% CI)		74, 89.3% (80.4–94.9%)		23, 56.5% (34.8–76.0%)		25, 72.0% (49.7–87.0%)

*Indication for ATV use: “preconception,” if already taking an atazanavir-containing regimen at the time of last menstrual period (LMP); “switch,” if atazanavir was commenced during this pregnancy in a woman already taking combination antiretroviral therapy (cART); “first-line,” if a first-line atazanavir-based regimen was initiated after the LMP.

**Total pregnancies: number of pregnancies with data available for each of the listed outcomes.

***Gastrointestinal side effects: nausea, vomiting, or diarrhoea.

^†^Transaminitis: AIDS Clinical Trial Group grade 1–4 transaminitis.

^††^Transaminitis in the “switch” exposure group presented for 24 women with normal transaminase levels at the time of switch.

^†††^ *n* refers to the number of live singleton/twin births in women who continued atazanavir until the time of delivery. % refers to the proportion of live births that were singleton or twin respectively.

^‡^ *n*: the number of events.

^‡‡^95% CI: 95% confidence interval calculated with robust standard errors.

^‡‡‡^ *N*: the number of cases with data available.

^§^Birth weight and gestation data are only presented for singleton deliveries.

^§§^IQR: interquartile range.

**Table 2:** Proportion of women achieving an HIV viral load <50  copies/mL by treatment regimen at time of delivery.

	*n* ∗	HIV viral load <50 copies/mL % (95% CI)
All regimens	122	80.3% (72.1–86.6%)
ATV 300/R throughout∗∗	101	83.2% (74.3–89.4%)
ATV 400/R escalated∗∗∗	11	72.7% (33.5–93.4%)
ATV 400 throughout^†^	7	50% (11.0–89.0%)
ATV >400^‡^	2	100% (15.8–100%)
ATV 400 (reduced to 300/R)^§^	1	100% (2.5–100%)

*n: number of pregnancies in which women were taking the specified regimen at the time of delivery.

∗∗ATV 300/R throughout: women were prescribed atazanavir 300 mg/ritonavir 100 mg at all times when an atazanavir based regimen was prescribed in the recorded pregnancy.

∗∗∗ATV 400/R escalated: women prescribed atazanavir 300 mg/ritonavir 100 mg initially; then dose increased to atazanavir 400 mg/ritonavir 100 mg in the third trimester.

^†^ATV 400 throughout: women prescribed atazanavir 400 mg without ritonavir throughout pregnancy.

^‡^ATV >400 two pregnancies, both occurring in the same woman, where a dose of atazanavir >400 mg was prescribed without ritonavir boosting.

^§^ATV 400 (reduced to 300/R): women prescribed atazanavir 400 mg without ritonavir at the start of pregnancy which was converted to atazanavir 300 mg/ritonavir 100 mg.
